# Comparison of Oil Content and Fatty Acid Profile of Ten New* Camellia oleifera* Cultivars

**DOI:** 10.1155/2016/3982486

**Published:** 2016-01-31

**Authors:** Chunying Yang, Xueming Liu, Zhiyi Chen, Yaosheng Lin, Siyuan Wang

**Affiliations:** Sericultural & Agri-Food Research Institute, Guangdong Academy of Agricultural Sciences/Key Laboratory of Functional Foods, Ministry of Agriculture/Guangdong Key Laboratory of Agricultural Products Processing, 133 Yihenglu, Dongguanzhuang, Tianhe District, Guangzhou 510610, China

## Abstract

The oil contents and fatty acid (FA) compositions of ten new and one wild* Camellia oleifera* varieties were investigated. Oil contents in camellia seeds from new* C. oleifera* varied with cultivars from 41.92% to 53.30% and were affected by cultivation place. Average oil content (47.83%) of dry seeds from all ten new cultivars was almost the same as that of wild common* C. oleifera* seeds (47.06%). New* C. oleifera* cultivars contained similar FA compositions which included palmitic acid (C16:0, PA), palmitoleic acid (C16:1), stearic acid (C18:0, SA), oleic acid (C18:1, OA), linoleic acid (C18:2, LA), linolenic acid (C18:3), eicosenoic acid (C20:1), and tetracosenoic acid (C24:1). Predominant FAs in mature seeds were OA (75.78%~81.39%), LA (4.85%~10.79%), PA (7.68%~10.01%), and SA (1.46%~2.97%) and OA had the least coefficient of variation among different new cultivars. Average ratio of single FA of ten artificial* C. oleifera* cultivars was consistent with that of wild common* C. oleifera*. All cultivars contained the same ratios of saturated FA (SFA) and unsaturated FA (USFA). Oil contents and FA profiles of new cultivars were not significantly affected by breeding and selection.

## 1. Introduction

The genus* Camellia* (Theaceae) is native to East Asia and comprises more than 200 woody evergreen species. Some species possess great economic value, particularly* C. sinensis*,* C. japonica*, and* C. oleifera*.* C. sinensis* is grown commercially mainly in tropical and subtropical regions for tea products;* C. japonica* is cultivated in temperate regions worldwide as ornamentals and its oil has a long history of traditional cosmetic usage in Japan as a protectant to maintain the health of skin and hair [1], while* C. oleifera* is planted mainly in China for high quality vegetable oil production.

*C. oleifera* Abel, also known as oil-tea camellia, an evergreen shrub or small tree in Camellia family, is one of the famous four woody oil plants (other three woody oil plants are oil olive, oil palm, and oil coconut). It can grow on barren land without fertilizers, start bearing fruits eight years after initial planting, and remain highly productive for 80 years.* C. oleifera* seed is mainly used for the production of edible oils, such as camellia oil, tea seed oil, or oil-tea camellia seed oil in China.

Camellia oil has much chemical composition in common with olive oil, with high amounts of oleic acid and linoleic acid and low saturated fats, and is often titled “Eastern Olive Oil.” Camellia oil was not only extensively used for cooking, but it has been used in traditional Chinese medicine and in cosmetics as well. In Chinese herbal medicine, it was considered as a superior nutritional dietary supplement that benefits the digestive system, reduces blood cholesterol, regulates the nervous system, and strengthens the immune system [2–5]. It was traditionally applied as a medicine for burning injury and new-born baby lotion in some place in China [6]. Modern medicinal research confirmed that camellia oil could decrease blood cholesterol content, provide resistance to oxidative stress [7], protect liver against carbon tetrachloride toxicity [8], and so forth. Camellia oil was recommended by the Food and Agriculture Organization of the United Nations as a high-quality, healthy vegetable oil because of its nutritional value and excellent storage qualities [9].

Camellia plant is mostly distributed in China, while Southeast Asia, Japan, and other countries own very little distribution. There is approximately 4 million hectare of* C. oleifera* Abel forest in China, spread in 14 provinces around Yangtze river basin and south Yangtze river area, mostly in Hunan, Jiangxi, and Guangxi [10]. In an effort to create more green land, increase farmer's income, and reduce China's dependence on imported oil, Chinese government agencies were setting policies to support the development of camellia oil industry. At present, the annual production of camellia oil is approximately 0.26 million tons, and this amount is expected to exceed 2.5 million tons by 2020, roughly equivalent to 15% to 25% of Chinese edible oil consumption [11].

In the past,* C. oleifera* Abel forests were mainly formed by nature or planted by human but nurtured by nature, and the yield of camellia oil per unit was very low, only 37.50~86.85 kg/ha for a long time in China [11]. For most crops, germplasm is the most important factor to raise output per unit. In order to increase camellia oil yield per unit, many attentions were paid to the breeding and selection of new* C. oleifera* varieties. More than 100 new strains of* C. oleifera* with high yield were bred by forest research scientists in the past ten years, their oil yields could reach 525~750 kg/ha; the highest could touch 1125 kg/ha. Then, oil yield per unit was the only criterion during the breeding of a new oil camellia variety in the past and little attention was paid to the oil quality. In some cases, the quality of crops was influenced by yields. In order to explore the features of new* C. oleifera* cultivars, oil contents and FA profiles of ten new* C. oleifera* cultivars cultivated in different provinces in China were investigated.

## 2. Materials and Methods

### 2.1. Samples and Reagents

The cultivars investigated were 10 new* C. oleifera* cultivars, that is, Changlin-3, Changlin-4, Changlin-18, Changlin-23, Changlin-27, Changlin-40, Changlin-53, Changlin-166, Xianglin-210 and Cenxi Ruanzhi, and a wild common* C. oleifera* sample. Seeds of wild common* C. oleifera* which were mixed seeds of many wild* C. oleifera* varieties were provided by a camellia oil plant and used as the control in this investigation. Except for the wild common* C. oleifera*, all other cultivars were bred in recent year and passed Chinese official variety certification and were being at large-scale cultivation stage. All these cultivars belong to* C. oleifera* species. They were planted in Anhui, Guangdong, Hubei, Jiangxi, and/or Zhejiang Province, which were most suitable areas for planting* C. oleifera* according to Camellia Forestry Plan drafted by the Ministry of Forestry, China [11].

The fruits of these camellia cultivars were collected from October to November 2012. A sample of each cultivar in each place was collected from at least five healthy looking plants. Harvesting was carried out at their fully mature stage when fruits began to split open and seeds were visible. Fruits were allowed to dry at room temperature for one week, and then seeds were removed from capsules. Seed samples of at least 1000 g were taken, and the dried samples were stored under −20°C until analysis.

All solvents used in the experiments were of HPLC grade and obtained from Merck (Germany). NaOH and Na_2_SO_4_ were purchased from Shanghai Chemical Reagent Plant (Shanghai, China).

### 2.2. Oil Content Determination

All sample seeds of different cultivars were powdered by a laboratory plant grinder, respectively. 10 g of three individual ground samples of dry seeds was weighed. Extraction of total* C. oleifera* oil was performed according to the published AOAC methods [12], using an automatic Soxhlet apparatus filled with 120 mL petroleum ether (60–90°C) as the extraction solvent. Residue was dried to constant weight in a drying oven at 105°C for 1 h and weighed.

### 2.3. FA GC-MS Analysis

FA methyl esters (FAMEs) were prepared by using NaOH/methanol method [13]. About 200 mg of oil was transferred into a ground glass stoppered test tube, treated with 5 mL 0.5 mol/L NaOH/methanol. The samples were mixed for 20 s on vortex mixer, shook once every 5 min, and left to react for 40 min at 60°C. The methyl esters were extracted using 5 mL *n*-hexane and the aqueous phase was discarded. The *n*-hexane extract containing the FAMEs was washed with water, dried using anhydrous sodium sulfate, and centrifuged at 2500 g for 5 min (Thermo Fisher, Biofuge Stratos, USA) for later analysis by GC-MS.

FA methyl esters were determined with gas chromatography-mass spectrometry equipped with a treated J&W DB-WAX polyethylene glycol column (30 m × 0.25 mm, 0.25 *μ*m; J&W Scientific, USA). FAMEs were separated and detected and their concentration was measured. The GC oven program was the following: 180°C (hold 5 min) to 230°C at 3°C/min (hold 15 min). The carrier gas (helium) flow rate was in constant flow mode at 1 mL/min. Split injection of 1 *μ*L with split ratio of 1 : 20 was carried out at 250°C with the purge valve on at 2.5 min. The mass spectrometer operated in electron impact and full-scan monitoring mode with transfer line at 280°C and ion source at 230°C; the solvent delay was set to 3 min. The results are expressed in relative percentage of each FA, calculated by normalization of the chromatographic peak area according to GB/T 17377-2008 (China). FA identification was made by analysis of MS spectra and indexing of NIST05aL spectral database.

### 2.4. Statistical Analyses

For triplicate experimental data, mean and standard deviations were calculated using Microsoft Excel 2010. The results were expressed as mean ± standard deviation. One way of variance analysis was applied for determining the significant difference at *p* < 0.05. The statistical analysis was conducted using SPSS 11 (SPSS Inc., Chicago, USA).

## 3. Results and Discussion

### 3.1. Oil Contents of Different* C. oleifera* Cultivars

Table 1 presents the oil contents of tested* C. oleifera* cultivars. There were significant differences in the oil contents of the seeds from different cultivars. The highest oil content was found in cultivar Changlin-166 (53.30%), while the lowest content was in cultivar Changlin-3 (41.92%). There were several reports on the factors affecting oil contents of camellia seeds. The most important factor was camellia species. Zhu et al. found that oil content order in five species investigated were* C. semiserrata* Chi (60%) >* C. vietnamensis* Huang and* C. oleifera* Abel (50%) >* C. gigantocarapa* Hu. and* C. oleifera* var. “nhge an” (40%) [14]. Gao et al. reported that maturity of camellia fruits had some effects on oil contents; oil contents in fully matured camellia seeds (dropped naturally) were 0.56~2.78% higher than those picked manually [15]. Moreover, geographic situation was also an important element remarkably affecting oil content of this crop.

Then, the average oil content (47.83%) of dry seeds from all new cultivars tested was almost the same as that of wild common* C. oleifera* seeds (47.06%), which was from many wild* C. oleifera* varieties formed by nature. The results implied that artificial breeding of* C. oleifera* did not affect oil content of seeds. Due to the fact that fruit yields of* C. oleifera* were significantly increased by artificial breeding, total camellia oil output would be remarkably enhanced. According to primary experimental cultivation results, estimated oil yield could be reached as high as 1125 kg/ha from present 37.5 kg/ha.

### 3.2. FA Composition of Different* C. oleifera* Cultivars

FA composition is the most important parameter for quality evaluation of edible oils. In many cases, FA profiles of edible oils are related to their prices. Oils with high unsaturated FA would be sold in higher prices because consumers thought that the higher the unsaturated FA, the healthier the edible oil. High price for extra virgin olive oil (EVOO) was mainly attributed to its high content of oleic acid and its richness in phenolic compounds, in addition to the hard and time-consuming tasks involved in the cultivation of olive trees, the harvesting of the fruits, and the extraction of the oil [16].

Several papers reported that camellia oil had very similar FA profile with olive oil; that is, oleic, linoleic, palmitic, and stearic acids were the major FAs [10, 15]. Because of the resemblance of FA composition to olive oil, camellia oil was titled “Eastern Olive Oil.” Previous investigations on FA composition of camellia oil mostly used seeds from the wild* C. oleifera* trees as the experimental material. FA profiles of ten artificial* C. oleifera* cultivars and one wild common* C. oleifera* sample were analyzed in this paper (shown in Table 2). All tested samples contained similar FA compositions which included PA (C16:0,), palmitoleic acid (C16:1), SA (C18:0,), OA (C18:1,), LA (C18:2,), linolenic acid (C18:3), eicosenoic acid (C20:1), and tetracosenoic acid (C24:1). Predominant FAs in tested samples were OA (75.78%~81.39%), LA (4.85%~10.79%), PA (7.68%~10.01%), and SA (1.46%~2.97%). Single FA had different coefficient of variation (CV) among different new cultivars. CV for OA, LA, PA, and SA content was 3.28%, 25.00%, 8.99%, and 22.6%, respectively. Wang et al. found that OA had the least CV in* C. chekiangoleosa* Hu cultivated in difference places [3, 4]. Therefore, OA ratio in oil could be served as one of the most suitable indices to evaluate the authenticity of camellia oil. Average values for every FA of all new cultivars were almost the same as those of the wild common variety. The results also were in accordance with reports on FA composition in the literature [17]. Contents of SFA, USFA, monounsaturated fatty acid (MUFA), and polyunsaturated fatty acid (PUFA) in the oil samples from seeds of analyzed* C. oleifera* cultivars were 9.78%~12.49%,  87.45%~90.17,  77.08%~82.78%, and 5.17%~11.27%. There were some difference in SFA and USFA contents between our results and data reported by Ma et al. [18]. In addition, all cultivars had almost the same total USFA content and SFA content though there were differences to some extent in MUFA and PUFA contents. All these results implied that FA profile of new cultivar was not significantly affected by artificial breeding and selection.

For the same cultivar, different cultivation places had slight influence on its FA composition. Changlin-3 cultivated in Zhejiang Province had higher OA (78.77%) and lower LA (8.39%) and PA (8.87%), while that cultivated in Anhui had lower OA (73.23%) and higher LA (12.52%) and PA (10.02%). Changlin-4 cultivated in Jiangxi Province had higher OA (79.73%) and lower LA (7.69%) and PA (8.79%), while that cultivated in Hubei had lower OA (74.25%) and higher LA (11.71%) and PA (10.08%). Effects of geographical positions on Changlin-18, -23, -27, -40, -53, and -166 cultivars were less than those on Changlin-3 and Changlin-4. Geographic and climatic differences could be attributed to this difference.

Changlin series of* C. oleifera* cultivars were bred by Research Institute of Subtropical Forestry of Chinese Academy of Forestry. Their oil yield per unit could reach 625~1125 kg/ha, 15~30 times as that of present wild* C. oleifera* populations. Although genetic distance and cluster analysis results showed that there was a great genetic diversity among the clones [19], the genetic distance (GD) was from 0.502 2 to 0.816 3, and, in the level of GD 0.35, their FA profiles were not markedly affected by genetic diversity. Maybe this is because all cultivars tested are belonging to one subspecies, that is,* C. oleifera*, and the main breeding technology for them was grafting. Zhu et al. found that there were some difference in FA composition among* C. semiserrata* Chi.,* C. gigantocarapa* Hu.,* C. oleifera* var. “nhge an”,* C. vietnamensis* Huang., and* C. oleifera* Abel [15]. Su et al. found that there were marked differences in the composition of FA among those populations from the same native Taiwanese Camellia species but different subspecies [20].

FA profile of camellia oil could also be influenced by maturity. During maturing stage of camellia seed, OA was obviously in a rising trend, PA and LA were in a downward trend, and SA was in a slight upward trend. Thus, camellia seeds should be collected after full maturation in order to produce camellia oil with high quality. All samples tested in the present study were collected at full maturtion stage, so effects of maturity on FA profiles could be neglected.

Edible vegetable oils from different plant origins had characterized FA profiles. Many adulteration analysis methods were developed based on vegetable oil FA profiles. Each type of oil has a different FA profile that determines the nature of its physicochemical and nutritional properties and also provides information on the quality of the oil. Chinese national standard for camellia oil (GB 11765-2003) specified data for SFA (7%~11%), OA (74%~87%), and LA (7%~14%). But in our present results, four cultivars (Changlin-23, Changlin-40, Changlin-166, and Xianglin-210) had lower LA content and one cultivar (Changlin-27) had higher SFA content than that in the standard. Previous report found that LA content in* C. gigantocarapa* Hu. and* C. chekiangoleosa* Hu were less than 7% [18]. In 132 different* C. oleifera* varieties tested, 35 samples had lower than 7.0% or higher than 14.0% LA content, while 19 samples had lower than 74% OA content [19]. Thus, it is the time to think about the adjustment on the SFA and LA content data in the national standard based on large sample analysis.

FA composition is closely related to lipid oxidation, product quality, and function of vegetable oils. Due to the high OA content, camellia oil provides health functions, such as lowering of blood pressure, cholesterol, and triglycerides, and thus is helpful in preventing cardiovascular diseases, cancer, hypertension, and autoimmune disorders. It is also of value in protecting the liver against carbon tetrachloride toxicity [8]. The presence of high amounts of OA gives camellia oil a higher stability and better health properties, and this is why it is known as “Eastern Olive oil.” Owing to its good organoleptic characteristics with high OA and low LA levels, camellia oil is a good material in cooking and in the food industry [20].

Most USFA is an essential substance but cannot be synthesized until the body is first supplied with food. An undersupply of USFA will result in dry skin and hair loss. USFA content is very high in camellia oil and, therefore, camellia oil can be easily digested and absorbed. LA, the most abundant USFA in camellia oil and an essential FA from the omega-6 group, is very important in developing and maintaining the nervous system and physiological functions in humans; furthermore, it is one of the key compounds of cell membranes, associated with brain function and neurotransmission, and plays an important role in the transference of O_2_ to blood plasma in the synthesis of hemoglobin [20, 21]. PA, a saturated fatty acid, could increase plasma cholesterol levels, while USFA decreases them. The desirable features of camellia oil depend on its low levels of SFA (<12%) and high levels of USFA (>88%). Data from present investigation suggested that oils from the new* C. oleifera* retained the excellent features of traditional camellia oil.

## 4. Conclusions

Oil contents in camellia seeds from new* C. oleifera* varied with cultivars from 41.92% to 53.30%. Average oil content (47.83%) of dry seeds from all ten new cultivars tested was almost the same as that of wild common* C. oleifera* seeds (47.06%). New* C. oleifera* cultivars contained similar FA compositions which included PA, palmitoleic acid, SA, OA, LA, linolenic acid, eicosenoic acid, and tetracosenoic acid. Predominant FAs in mature seeds were OA (75.78%~81.39%), LA (4.85%~10.79%), PA (7.68%~10.01%), and SA (1.46%~2.97%) and OA had the least coefficient of variation among different new cultivars. All tested samples contained the same ratios of SFA and USFA. Oil contents and FA profiles of new cultivars were not significantly affected by artificial breeding and selection.

## Figures and Tables

**Table 1:** Oil contents of different *C. oleifera* cultivars.

*C. oleifera* cultivar	Oil content (%)
Changlin-3	41.92 ± 8.07^c^
Changlin-4	43.30 ± 7.93^c^
Changlin-18	51.45 ± 3.59^ab^
Changlin-23	51.85 ± 1.90^ab^
Changlin-27	44.64 ± 0.92^c^
Changlin-40	50.22 ± 4.70^b^
Changlin-53	46.83 ± 1.50^c^
Changlin-166	53.30 ± 2.55^a^
Xianglin-210	49.20 ± 0.03^b^
Cenxi Ruanzhi	44.36 ± 0.13^c^

Average of new cultivars	47.83 ± 5.61^bc^

Wild common *C. oleifera*	47.06 ± 0.77^bc^

^*∗*^Means without a common letter in the same column differ (*p* > 0.05).

**Table 2:** FA composition (%) of different *C. oleifera* cultivars.

Cultivar	C16:0	C16:1	C18:0	C18:1	C18:2	C18:3	C20:1	C24:1	∑SFA	∑MUFA	∑PUFA	∑USFA
Changlin-3	9.48 ± 0.58^ab^	0.22 ± 0.11	1.71 ± 0.43^bc^	76.03 ± 2.77^a^	10.79 ± 2.15^a^	0.48 ± 0.29	0.70 ± 0.29	0.12 ± 0.06	11.38 ± 0.17^a^	77.26 ± 2.53^a^	11.27 ± 2.32^a^	88.53 ± 0.22^a^
Changlin-4	9.50 ± 0.66^ab^	0.22 ± 0.06	1.46 ± 0.35^c^	76.89 ± 2.75^a^	9.70 ± 2.01^a^	0.66 ± 0.21	0.97 ± 0.12	0.17 ± 0.05	11.18 ± 0.34^a^	78.40 ± 2.54^a^	10.36 ± 2.18^a^	88.75 ± 0.36^a^
Changlin-18	9.17 ± 0.42^b^	0.25 ± 0.03	1.98 ± 0.18^b^	77.46 ± 1.85^a^	9.44 ± 1.48^a^	0.43 ± 0.08	0.78 ± 0.01	0.10 ± 0.01	11.36 ± 0.23^a^	78.69 ± 1.82^a^	9.87 ± 1.57^ab^	88.56 ± 0.25^a^
Changlin-23	8.30 ± 0.15^bc^	0.24 ± 0.17	2.59 ± 0.38^b^	80.86 ± 0.75^a^	6.47 ± 0.48^d^	0.30 ± 0.05	0.74 ± 0.08	0.09 ± 0.01	11.15 ± 0.32^a^	82.04 ± 0.60^a^	6.76 ± 0.52^c^	88.80 ± 0.32^a^
Changlin-27	10.01 ± 0.15^a^	0.22 ± 0.04	2.24 ± 0.02^b^	75.78 ± 0.50^a^	9.92 ± 0.39^a^	0.46 ± 0.05	0.85 ± 0.10	0.12 ± 0.01	12.49 ± 0.09^a^	77.08 ± 0.34^a^	10.37 ± 0.44^a^	87.45 ± 0.10^a^
Changlin-40	8.86 ± 0.12^b^	0.16 ± 0.03	2.31 ± 0.35^b^	80.32 ± 1.31^a^	6.95 ± 1.21^d^	0.31 ± 0.07	0.68 ± 0.12	0.08 ± 0.03	11.36 ± 0.32^a^	81.33 ± 1.14^a^	7.26 ± 1.28^b^	88.59 ± 0.31^a^
Changlin-53	7.68 ± 0.85^c^	0.55 ± 0.67	1.89 ± 0.12^b^	81.01 ± 1.04^a^	7.09 ± 1.08^d^	0.36 ± 0.05	0.90 ± 0.23	0.12 ± 0.03	9.78 ± 0.81^a^	82.71 ± 1.85^a^	7.45 ± 1.05^b^	90.17 ± 0.80^a^
Changlin-166	8.45 ± 0.25^bc^	0.14 ± 0.02	2.32 ± 0.20^b^	79.71 ± 2.39^a^	6.85 ± 0.47^d^	1.11 ± 1.48	0.71 ± 0.08	0.36 ± 0.49	10.97 ± 0.24^a^	81.02 ± 1.82^a^	7.96 ± 1.78^b^	88.98 ± 0.25^a^
Xianglin-210	8.61 ± 0.03^bc^	0.18 ± 0.02	2.97 ± 0.02^a^	81.39 ± 0.07^a^	4.85 ± 0.02^e^	0.32 ± 0.02	0.94 ± 0.03	0.11 ± 0.01	11.96 ± 0.22^a^	82.78 ± 0.15^a^	5.17 ± 0.04^c^	87.95 ± 0.19^a^
Cenxi Ruanzhi	9.55 ± 0.04^ab^	0.22 ± 0.02	1.67 ± 0.02^bc^	77.64 ± 0.06^a^	8.77 ± 0.04^b^	0.55 ± 0.02	0.97 ± 0.02	0.14 ± 0.02	11.47 ± 0.16^a^	79.11 ± 0.14^a^	9.32 ± 0.06^ab^	88.43 ± 0.20^a^

Average value of new cultivars	8.89 ± 0.80^bc^	0.25 ± 0.24	2.08 ± 0.47^b^	78.75 ± 2.58^a^	8.16 ± 2.04^c^	0.51 ± 0.52	0.81 ± 0.16	0.14 ± 0.17	11.19 ± 0.74^a^	80.07 ± 2.54^a^	8.67 ± 2.17^ab^	88.74 ± 0.75^a^

Common *C. oleifera*	9.67 ± 0.02^ab^	0.16 ± 0.01	1.87 ± 0.03^b^	77.59 ± 0.05^a^	8.96 ± 0.03^b^	0.28 ± 0.02	0.90 ± 0.02	0.12 ± 0.02	11.82 ± 0.19^a^	78.90 ± 0.12^a^	9.24 ± 0.05^ab^	88.14 ± 0.17^a^

MUFA: monounsaturated fatty acids; PUFA: polyunsaturated fatty acids; SFA: saturated fatty acids; USFA: unsaturated fatty acids.

^*∗*^Means without a common letter differ (*p* > 0.05).

## Abbreviations

### Abbreviations

FA: Fatty acid
PA: Palmitic acid
SA: Stearic acid
OA: Oleic acid
SFA: Saturated FA
USFA: Unsaturated FA
FAMEs: FA methyl esters
EVOO: Extra virgin olive oil
CV: Coefficient of variation
MUFA: Monounsaturated fatty acid
PUFA: Polyunsaturated fatty acid
GD: Genetic distance.

## References

[1] Jung E., Lee J., Baek J. (2007). Effect of *Camellia japonica* oil on human type I procollagen production and skin barrier function. *Journal of Ethnopharmacology*.

[2] Cheng Y.-T., Wu S.-L., Ho C.-Y., Huang S.-M., Cheng C.-L., Yen G.-C. (2014). Beneficial effects of camellia oil (*Camellia oleifera* Abel.) on ketoprofen-induced gastrointestinal mucosal damage through upregulation of HO-1 and VEGF. *Journal of Agricultural and Food Chemistry*.

[3] Wang K. L., Cao F. L., Yao X. H. (2011). Chemical composition of fatty acid from *Camellia chekiangoleosa* Hu. *Journal of Nanjing Forest University (Natural Science Edition)*.

[4] Wang Y., Sun D., Chen H., Qian L., Xu P. (2011). Fatty acid composition and antioxidant activity of tea (*Camellia sinensis* L.) seed oil extracted by optimized supercritical carbon dioxide. *International Journal of Molecular Sciences*.

[5] Feng X., Zhou Y. Z. (1996). Influences of feeding tea seed oil, corn oil and fish oil on immune status in mice. *Acta Nutrimenta Sinica*.

[6] Lin C. Y., Cheng B. (2011). Effects of topical application of camellia oil on the development of allergic contact dermatitis. *Chinese Journal of Dermatology*.

[7] Lee C.-P., Yen G.-C. (2006). Antioxidant activity and bioactive compounds of tea seed (*Camellia oleifera* Abel.) oil. *Journal of Agricultural and Food Chemistry*.

[8] Lee C.-P., Shih P.-H., Hsu C.-L., Yen G.-C. (2007). Hepatoprotection of tea seed oil (*Camellia oleifera* Abel.) against CCl4-induced oxidative damage in rats. *Food and Chemical Toxicology*.

[9] Zhu X. Y., Lin H. M., Chen X., Xie J., Wang P. (2011). Mechanochemical-assisted extraction and antioxidant activities of kaempferol glycosides from *Camellia oleifera* Abel. meal. *Journal of Agricultural and Food Chemistry*.

[10] Li S. F., Zhu X. R., Zhang J. H., Li G. Y., Su D., Shan Y. (2012). Authentication of pure camellia oil by using near infrared spectroscopy and pattern recognition techniques. *Journal of Food Science*.

[11] (2009). *National Oil Camellia industry Development Plan (2009–2020)*.

[12] AOAC (2000). *Official Methods of Analysis of the Association of Official Analytical Chemists*.

[13] O'fallon J. V., Busboom J. R., Nelson M. L., Gaskins C. T. (2007). A direct method for fatty acid methyl ester synthesis: application to wet meat tissues, oils, and feedstuffs. *Journal of Animal Science*.

[14] Zhu Y., Wang X. Y., Ma J. L., Ye H., Zhong H. Y. (2013). Oil content in seed and fatty acid composition in oil of different species of *Camellia oleifera*. *Nonwood Forest Research*.

[15] Gao W., He X. S., Sun Y., Zhan Z. Y., Huang J. J., Lei X. L. (2013). Effects of different harvest methods on oil content in *Camellia oleifera*. *Nonwood Forest Research*.

[16] Lerma-García M. J., Ramis-Ramos G., Herrero-Martínez J. M., Simó-Alfonso E. F. (2010). Authentication of extra virgin olive oils by Fourier-transform infrared spectroscopy. *Food Chemistry*.

[17] Yuan J., Wang C., Chen H., Zhou H., Ye J. (2013). Prediction of fatty acid composition in *Camellia oleifera* oil by near infrared transmittance spectroscopy (NITS). *Food Chemistry*.

[18] Ma J., Ye H., Rui Y., Chen G., Zhang N. (2011). Fatty acid composition of *Camellia oleifera* oil. *Journal of Consumer Protection and Food Safety*.

[19] Lin P., Yao X. H., Wang K. L., Zheng T. T., Teng J. H. (2010). Identification and genetic analysis of *Camellia oleifera* Changlin series superior clones by SRAP molecular marker. *Journal of Agricultural Biotechnology*.

[20] Su M. H., Shih M. C., Lin K.-H. (2014). Chemical composition of seed oils in native Taiwanese *Camellia* species. *Food Chemistry*.

[21] Kaya-Dagistanli F., Tanriverdi G., Altinok A., Ozyazgan S., Ozturk M. (2013). The effects of alpha lipoic acid on liver cells damages and apoptosis induced by polyunsaturated fatty acids. *Food and Chemical Toxicology*.

